# Multi-Pathway Study for Oxaliplatin Resistance Reduction

**DOI:** 10.3390/cimb47030172

**Published:** 2025-03-04

**Authors:** Tong Ye, Chen Wu, Jintong Na, Xiyu Liu, Yong Huang

**Affiliations:** 1 State Key Laboratory of Targeting Oncology, National Center for International Research of Bio-Targeting Theranostics, Guangxi Key Laboratory of Bio-Targeting Theranostics, Guangxi Medical University, Nanning 530021, China; yetong0906@outlook.com; 2 Collaborative Innovation Center for Targeting Tumor Diagnosis and Therapy, Guangxi Medical University, Nanning 530021, China; w18866143808@163.com; 3 Guangxi Talent Highland of Major New Drugs Innovation and Development, Guangxi Medical University, Nanning 530021, China; najintong@sr.gxmu.edu.cn

**Keywords:** oxaliplatin, cancer therapy, drug resistance, anticancer mechanism, combination therapy

## Abstract

Chemotherapy for cancer frequently uses platinum-based medications, including oxaliplatin, carboplatin, and cisplatin; however, due to their high systemic toxicity, lack of selectivity, drug resistance, and other side effects, platinum-based medications have very limited clinical application. As a first-line medication in antitumor therapy, oxaliplatin must be administered to minimize side effects while achieving anticancer objectives. A new CDC7 inhibitor called XL413 has demonstrated promising antitumor therapeutic effects in a variety of malignant tumors and may have anticancer properties. This offers a fresh viewpoint on how to lessen oxaliplatin resistance and, specifically, increase the potency of already prescribed anticancer therapies. In this paper, the current developments in anticancer therapy are discussed, along with the many mechanisms of oxaliplatin’s antitumor effects, clinical treatment challenges, and related approaches. We conducted more research on oxaliplatin resistance that arose during chemotherapy and searched for ways to lessen it in order to enhance its chemotherapeutic performance. Ultimately, we studied how distinct resistance routes relate to one another. Meanwhile, XL413, a novel CDC7 inhibitor, offers a perspective on the possibilities for developing treatment approaches for this innovation point. The search terms “Oxaliplatin, XL413, drug resistance, cancer treatment,” etc., were applied in the X-MOL and PubMed databases for this review’s literature search. Boolean logic was then employed to maximize the search approach. These databases can offer thorough research data and cover a broad range of biological publications. Excluded publications were works of low relevance, duplicates, or those with insufficient information. The mechanism of oxaliplatin’s anticancer effect, oxaliplatin resistance and its amelioration, and the role of XL413 in oxaliplatin treatment were the main topics of the 140 publications that were ultimately included for analysis.

## 1. Introduction

In the 1960s, Rosenberg, a professor of biophysics in the United States, accidentally discovered that platinum compounds could inhibit bacterial division, and then reported that cisplatin had potential anticancer activity, which opened the prelude to the treatment of malignant tumors with platinum-based drugs [1]. Common malignant tumors, including those of the lung, bladder, ovaries, cervix, esophagus, stomach, colorectal, and head and neck regions, are treated with platinum-based chemotherapy drugs, which are the most commonly used chemotherapy agents in clinical settings [2]. Given its distinct mechanism and extensive clinical uses, oxaliplatin, a third-generation platinum-based chemotherapeutic drug, has become known as a significant option for the treatment of a variety of malignancies [3].

XL413 is a confident CDC7 inhibitor that is highly selective towards DDK and can efficiently inhibit DDK. This inhibition may lead to apoptosis in a range of cancer cell types, with no effect on healthy cells [4]. Thus, it makes sense to utilize XL413 in conjunction with chemotherapeutic medicines based on its capacity to target tumor cells, prevent the progression of cancer, and eliminate cancer cells [5].

Platinum medicines are first-line chemotherapy medications, and their resistance has historically been the largest barrier to chemotherapy’s effectiveness. Reducing platinum drug resistance can minimize adverse effects, lower systemic toxicity, and enhance prognosis. Additionally, the prognosis for cancer may be improved when combined with the CDC7 inhibitor XL413.

This paper reviews the most recent developments in the mechanism and research on oxaliplatin, the third generation of platinum-based anticancer medications, and suggests various approaches to combat drug resistance. Moreover, a novel treatment plan for cancer that involves oxaliplatin and the CDC7 inhibitor XL413 was released.

## 2. Information and Methodology

### 2.1. Sources of Information

#### 2.1.1. Searchers and Time of the Search

The search was conducted in September 2024 by the first author.

#### 2.1.2. Timeframe for Searching the Literature

The timeframe was from January 2000 to February 2025, with a small amount of distantly related literature included for review.

#### 2.1.3. Search Databases

The X-MOL database and PubMed database were used.

#### 2.1.4. Type of Literature Searched

Research papers and reviews were searched.

#### 2.1.5. Search Terms

The database search terms are ‘Oxaliplatin, XL413, CDC7 inhibitor, Drug resistance, Cancer treatment’. The search terms are ‘Oxaliplatin, XL413, CDC7 inhibitor, Drug resistance, Cancer treatment’, etc. The search terms are connected by the Boolean logical operators ‘OR’ and ‘AND’.

#### 2.1.6. Search Strategy

The search strategy of X-MOL for the Web of Science and PubMed databases, for example, is shown in Table 1.

### 2.2. Criteria for Inclusion and Exclusion

#### 2.2.1. Criteria for Inclusion

Journal articles, dissertations, and other works with adequate proof and unbiased findings comprise the included literature. ② The study focuses on the use of XL413 and oxaliplatin in cancer treatment.

#### 2.2.2. Criteria for Exclusion

Exclusion criteria were as follows: ① literature that is not very relevant to the review’s topic; ② literature that has been published frequently; ③ literature with thesis opinions that are too old; ④ literature that contains research that is devoid of experimental data or methodological specifics.

#### 2.2.3. Evaluation of the Literature Quality and Data Extraction

**Figure 1:**
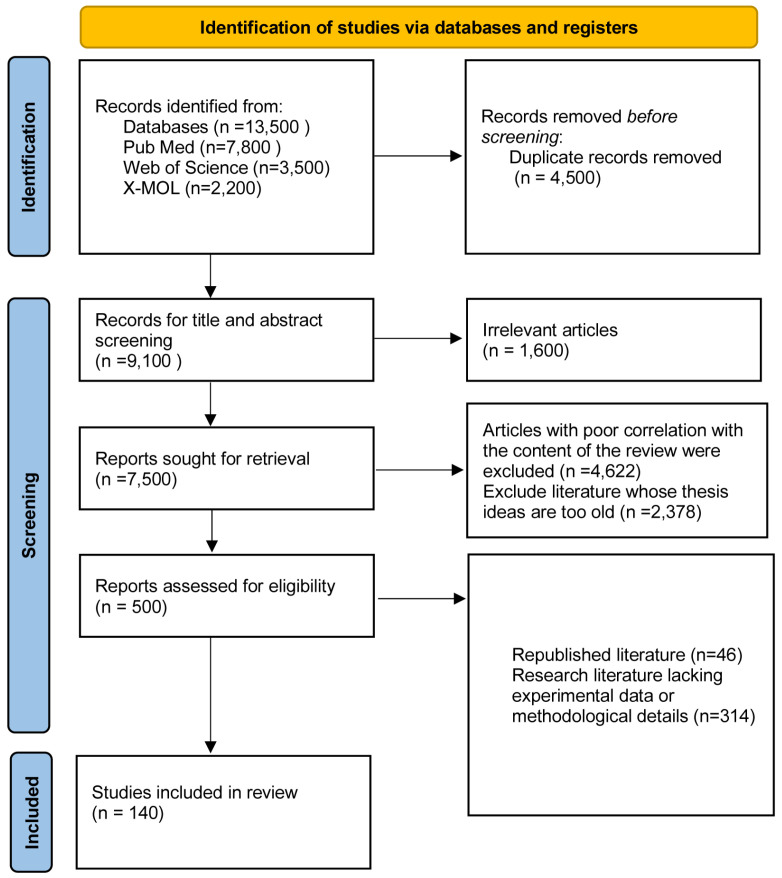
Flowchart of the literature quality evaluation and data extraction.

Based on the preliminary screening, a total of 13,500 papers were searched; duplicates were eliminated, leaving 9100 documents; documents with irrelevant abstracts and titles were eliminated, leaving 500 documents; and based on relevant matches, finally, 140 documents that fulfilled the review requirements were included (Figure 1). The actual number may differ slightly depending on certain search criteria and database updates, but the results are based on search logic and bibliometric studies of the PubMed and X-MOL databases.

## 3. Development of First-Line Pt Drugs

Chemotherapy is an effective cancer therapy approach [6,7]. Prior to the 1960s, all cancer medicines were made entirely of organic substances [8].

Cisplatin, a straightforward coordination molecule with anticancer capabilities, was accidentally found in the late 1960s, and was discovered to have the cytostatic capability to prevent the growth of germs. New avenues for cancer chemotherapy have been made possible by this discovery.

Anticancer drugs based on platinum, like cisplatin [9], carboplatin [10], and oxaliplatin [11], have been used widely in clinical practice due to their obvious therapeutic efficacy and well-defined mechanisms of action.

Cisplatin is a first-generation platinum-based anticancer medication that clearly operates on a range of cancers, including colorectal, breast, and ovarian cancers [12]. On the other hand, cisplatin is a non-specific chemotherapeutic therapy that kills tumor cells but also has systemic toxicity [13]. Therefore, long-term use of cisplatin can cause substantial harm to normal tissues, and platinum-based anticancer medicines have serious adverse responses, including dose-limiting toxicity, particularly nephrotoxicity, neurotoxicity, ototoxicity, and myelosuppression [14]. A number of techniques, involving liposome encapsulation [15], nanomaterial carrier drug delivery [16], and bioconjugation that targets the highly expressed protein portion of the tumor [17], have been used to prevent damage to normal tissues due to clinical first-line Pt drugs, like cisplatin, which have significant therapeutic effects on tumor tissues.

Consequently, the first-generation platinum-based chemotherapeutic agent, cisplatin, which took more than 10 years to reach clinics, formed the basis for the invention of the second-generation platinum-based medicine carboplatin.

As carboplatin contains bidentate cyclobutane dicarboxylic acid ligands and has a lower hydration rate than cisplatin, it has superior biosafety and much lower systemic toxicity, including hepatotoxicity, nephrotoxicity, neurotoxicity, and ototoxicity [18]. Carboplatin can be used as a high-dose chemotherapy for aggressive cancers because of its minimal toxicity. However, a major problem with platinum treatment is platinum resistance [19], wherein resistance to carboplatin and cisplatin gradually builds up throughout therapy.

As it lacks cross-resistance with either cisplatin or carboplatin, the third-generation platinum-based clinical drug oxaliplatin has a similar mechanism and action to cisplatin. This implies that oxaliplatin and cisplatin can operate in tandem to provide clinical anticancer therapy, which is why it has been used extensively in clinical trials [20,21]. None of the platinum-based anticancer medications have achieved worldwide clinical use despite several attempts to develop them and lessen their negative effects (Table 2).

## 4. Molecular Mechanism of Oxaliplatin

Over the last 20 years, thousands of platinum complexes have been established, and over 25 of them have advanced to the clinical trial stage, which is devoted to creating analogues with less toxicity and no cross-resistance. Although oxaliplatins’ precise mode of action is unknown, it is believed that restriction of DNA synthesis is the cause of platinum compounds’ cytotoxicity. By producing cross-linking within the DNA strand, the platinum-based biological oxaliplatin prevents DNA synthesis.

The intrastrand platinum–DNA adduct, which is produced by crosslinks between activated platinum and specified base sequences—specifically, two adjacent guanine residues—is oxaliplatins’ main cytotoxic lesion. A diaminocyclohexane (DACH) carrier ligand serves as the basis for the platinum complex oxaliplatin.

Both the increased activity and the lack of cross-resistance between oxaliplatin and cisplatin are thought to be caused by the massive DACH carrier ligand for oxaliplatin [22]. By blocking or limiting the binding of particular damage repair proteins (such as mismatch repair enzyme complexes), DACH ligands may also hinder DNA repair by diminishing the replication bypass of platinum–DNA adducts [22,23]. Oxaliplatin has been shown to alter DNA integrity and trigger apoptosis due to these or other contributing factors, which may also be connected to the drug’s mechanism of action [24,25].

**Figure 2:**
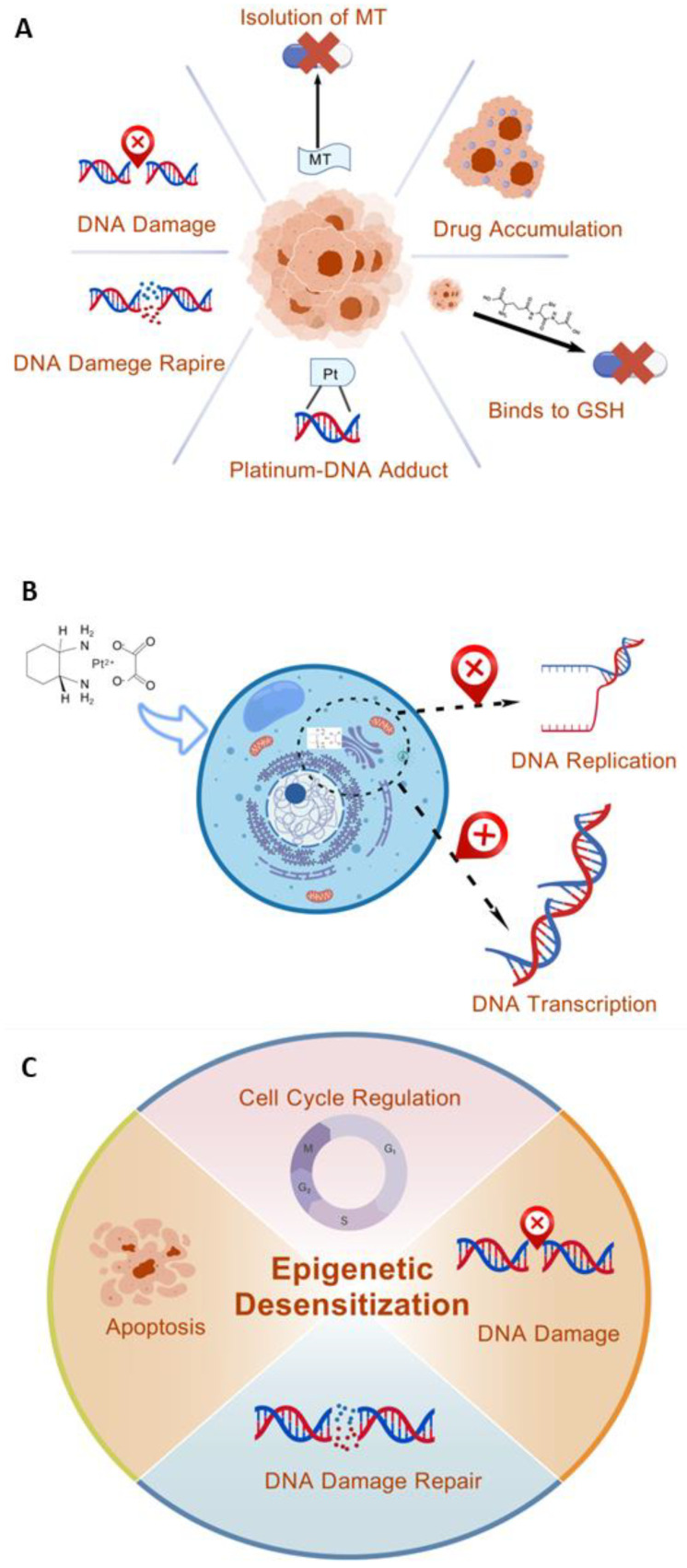
(**A**) Mechanisms involved in the development of resistance of tumor cells to all platinum compounds. (**B**) Process and results of oxaliplatin binding to DNA after entering the cell. (**C**) Epigenetic mechanisms of desensitization pathways for cancer chemotherapy resistance. Created using BioGDP.com [35].

Tumor cells become resistant to all platinum compounds through a variety of strategies, including the following: enhanced tolerance to platinum–DNA adducts, upgraded excision repair of platinum–DNA adducts, decreased cellular drug accumulation, and drug inactivation by binding to glutathione or segregation involving metallothionine [22,26] (Figure 2A). Though they have been shown to cause oxaliplatin resistance, mismatch repair errors and rose replication bypass have also been identified as mechanisms of cisplatin or carboplatin resistance [22,27]. Hence, it is essential to comprehend how this platinum medication produces its anticancer effects in order to have a better understanding of the pathways underlying oxaliplatin resistance.

[oxalate(2-)-o, 0] Oxaliplatin-cyclohexanediamine-n, 0 [1R, 2R]platinum-(II)} refers to a family of chemotherapeutic drugs that also contains carboplatin and cisplatin. (1R, 2R) Cyclohexane-1,2-diamine (R,R-DACH), a bidentate ligand, takes the place of two amine ligands in oxaliplatin. Compared to its alternatives, this structural variation confers a different spectrum of activity and activates different pathways for detecting cellular damage [28]. Intravenous administration of oxaliplatin is utilized. In terms of pharmacokinetics, it is distinguished by a brief initial period of distribution and a lengthy final period of drug elimination, which mainly occur in the kidney 48 h after administration [29].

Oxaliplatin attributes itself to nucleophilic cells—which are primarily DNA-dominated, yet they also contain RNA and proteins—as it enters the cell [29]. By creating an intrastrand adduct between two nearby guanine residues or guanine and adenine, it competes with DNA replication and transcription as a DNA-interacting agent (Figure 2B). At equimolar concentrations, oxaliplatin generates greater cytotoxicity than cisplatin due to its wider structure, but it generates fewer DNA adducts [30].

Based on some studies, oxaliplatin and other platinum compounds may result in the production of free radicals, which can damage DNA oxidatively. Notably, oxaliplatin’s efficacy is linked to the generation of reactive oxygen species, which enhance necrosis [31]. Several studies have demonstrated that epigenetic mechanisms directly contribute to cancer chemoresistance, typically by deregulating genes involved in cell cycle regulation, apoptosis, DNA damage response, and DNA repair pathways (Figure 2C). Furthermore, it has been reported that acquired chemotherapy resistance arises as a result of chemotherapy’s ability to selectively activate drug-sensitive genes that are epigenetically silenced and found in cellular subpopulations; however, little is known about the epigenetic processes underlying oxaliplatin resistance [32]. Although oxaliplatin has a broad spectrum of anticancer activity and is now the third Pt-based anticancer therapy in clinical use worldwide, acquired resistance is still a major obstacle to oxaliplatin therapy [33]. Despite the fact that there is much research to be conducted, clarifying the molecular mechanisms behind the resistance phenomenon is essential, as they are the main explanation of therapy failure and tumor formation [34].

Hepatocellular carcinoma (HCC) can be effectively treated with chemotherapy based on oxaliplatin; nonetheless, oxaliplatin resistance, either acquired or primary, continues to be a major clinical problem.

## 5. Strategies to Enhance Platinum-Based Anticancer Efficiency and Reduce Systemic Resistance

To try to combat or lessen the negative effects of platinum (II)-based therapy resistance, new platinum-based medications have been produced [36]. Discovering and creating multi-acting Pt(IV) complexes with potential anticancer effects is the newest finding in this field [37]. Axial ligands can either be physiologically responsive or biologically inactive, like hydroxyl or halo ligands [38,39,40].

A vital process in organic synthesis is the oxidation of primary and secondary alcohols to supply the appropriate carbonyl molecules [41]. Chromium (VI) oxidizing agents are typically used in conventional processes for executing such reactions [42]; however, powerful catalytic strategies utilizing clean oxidants like O_2_ and H_2_O_2_ have been researched from the perspective of preventing environmental issues. It was recently shown that transition metals can catalytically oxidize alcohols when oxidants occur [43,44,45]. Furthermore, the establishment of guidelines for the effective design of catalysts has been made possible by the relationship between the structural chemistry of the catalyst and the characteristics of the reactants in a particular environment. Substrate effects are one of the primary factors that greatly influence catalytic activity. Additionally, research on Pt(IV) complexes’ potential anticancer activity and catalytic oxidation efficiency might provide novel opportunities for improving platinum’s effectiveness in fighting cancer and reducing platinum resistance.

### 5.1. Glutathione-Based Strategies to Reduce Oxaliplatin Resistance

#### 5.1.1. Improving Plasma Half-Life and Tumor Selection

**Figure 3:**
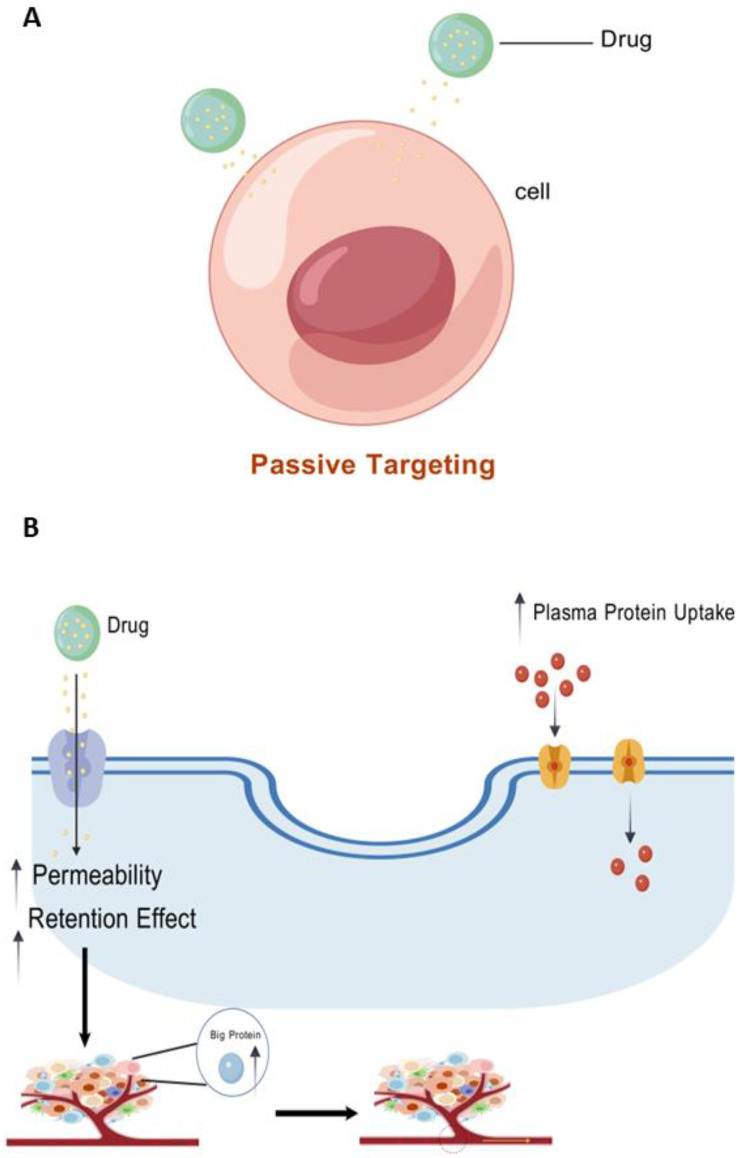
(**A**) Passive targeting of albumin. (**B**) Platinum (IV) complexes based on oxaliplatin and having a maleimide portion are transported to tumor tissue after binding to the unique free sulfhydryl group at position 34 of albumin, accumulating by two mechanisms. Created using BioGDP.com [35].

Phase III clinical studies for sartoplatin, the most promising platinum (IV) produced to date, were unsuccessful, probably due to its rapid decrease in erythrocytes. Therefore, increasing the plasma half-life and tumor selection of this class of compounds—for example, by passive targeting of albumin—is a way to potentially improve them [46,47] (Figure 3A). It has been reported that a platinum (IV) complex based on oxaliplatin that contains a maleimide portion exhibits strong anticancer activity in vivo [48]. The sole free sulfhydryl group at position 34 of albumin is rapidly and selectively reacted with by the prodrug’s maleimide. The two following processes then transport the covalently attached medication to the tumor tissue, where it builds up (Figure 3B):

(1) It has been demonstrated that cancer cells utilize albumin as a source of amino acids, which explains why they exhibit a greater uptake of this plasma protein [49,50].
(2) Open-window vascularization and decreased lymphatic drainage are caused by enhanced permeability and retention (EPR) effects, which enrich macromolecules in malignant tissues as a result of disarray [51].

Applying more biologically active substances to either or both of the axial sites of the platinum (IV) complex, which are liberated during reduction, is a better option. This allows for the synergist to be applied to the platinum (II) release sites that are mediated by reduction. The attachment of up to three distinct active components to platinum cores has also been reported, and this strategy has been successfully studied [52,53]. To selectively prevent acquired resistance mechanisms, for instance, psychologically active ligands can be applied.

#### 5.1.2. Inhibition of Glutathione Synthesis in Malignant Tissues

Increasing intracellular or extracellular glutathione levels in cancer cells or tumor microenvironments has been associated with the expulsion of biothiols (glutathione, cysteine), for example, by fibroblasts linked to cancer [8,53]. Platinum (II) drugs, such as oxaliplatin, may be turned inactive, or their efflux may be enhanced by both passive and glutathione-transferase (GST)-catalyzed adduct formation [54,55]. There have been encouraging reports on cisplatin platinum (IV) prodrugs utilizing ethacrynic acid, a GSTase inhibitor, as an axial ligand [56,57].

The inhibition of GSH synthesis in malignant tissues offers a more thorough strategy to steer clear of GSH-based platinum resistance mechanisms, as there is currently no such treatment approach for oxaliplatin.

#### 5.1.3. Reduced Drug Intake or Increased Drug Exportation

The enzyme that inhibits the rate of glutathione biosynthesis is glutamate-cysteine ligase (GCL) [58]. Despite being an energetic, selective, and irreversible inhibitor of glutamate-cysteine ligase (GCL), 1-buthionine-sulfoximine (BSO) has demonstrated the capacity to resensitize cancer cells that are resistant to adriamycin and cisplatin to drug therapy. Additionally, in a mouse model of ovarian cancer, BSO has demonstrated exceptional synergism with platinum-based medications [59]. Furthermore, BSO is undergoing clinical research as a sensitizer and has shown strong anticancer activity when combined with the alkylating drugs melphalan and arsenic trioxide [60,61]. This research indicates that platinum (IV) prodrugs are considerably less impacted by associated resistance mechanisms, as seen by the fact that they have substantially lower resistance factors under various resistance patterns.

Regarding oxaliplatin resistance, HCT116 (oxaliplatin-resistant group) cells indicated a greatly reduced intracellular accumulation of oxaliplatin in contrast with the original cell line. This suggests that decreased drug uptake or enhanced export are at least partially responsible for the acquired platinum resistance phenotype.

#### 5.1.4. Support for GSH Conversion Enhancements

Oxaliplatin 1-buthionine-s,r-sulfinamide (BSO), an antagonist of glutamate cysteine ligase, which co-synthesizes BSO-OxOAc, is the basis for the release of one platinum (IV) prodrug. The concept that variations in platinum accumulation between parental and drug-resistant HCT116 cells grow over time [62] provides strong evidence for the critical role of oxaliplatin efflux structures, such as the ABCC family of GSH-binding transporter proteins [58,63,64].

Extracellular reduction should be combined with intracellular reduction activation to target the platinum (IV) prodrug system, since this promotes more effective absorption of released platinum (II) molecules. According to the aforementioned information, biothiols strongly inactivate free oxaliplatin, probably as a result of the formation of extracellular connectors, whereas BSO-OxOAc is shielded from extracellular inactivation since it is a prodrug.

The unraveling of the complex redox balance in this resistance paradigm was reinforced by the strong resistance of HCT116/OxR cells to oxaliplatin in the clonal context. Oxaliplatin is highly reactive to the reducing (bio)thiol n-acetylcysteine (NAC) and to reducing drugs at greater concentrations. Though BSO-OxOAc alone diminished cellular GSH content significantly, when combined with the reducing agent, it significantly decreased cellular GSH content. Conversely, oxaliplatin did not change GSH content in in vitro studies. This effect was notably greater in the oxaliplatin-resistant subline, especially in the combination scenario, confirming the notion that increased GSH conversion is a factor in the resistance phenotype.

### 5.2. In Vivo Anticancer Activity

The way the immune system contributes to oxaliplatin’s anticancer effects is widely known. In addition to oxaliplatin-induced DNA platinization and DNA strand breaks, adaptive immune responses are essential for anticancer efficacy, as animals devoid of T cells are typically not susceptible to treatment.

In contrast, oxaliplatin accumulation was much higher in all evaluated non-tumor tissues compared to tumor tissues, according to a study using oxaliplatin derivatives in xenograft tumors of immunocompetent mice [48]. Therefore, improved tumor-specific accumulation and platinum (IV) prodrug activation may help decrease the side effects of oxaliplatin therapy.

### 5.3. Discovery of Drug Resistance Genes and Prediction of Drug Resistance

#### 5.3.1. Discovery of Drug Resistance Genes

##### Platinum Prodrug Design and Its Compounds

The development of acquired resistance phenotypes and severe side effects limits the sensitive application of platinum (II)-centered anticancer metallodrugs like carboplatin, oxaliplatin, and cisplatin. Platinum (IV) premedicine complexes may be a promising alternative in this regard. The distinctive characteristics of platinum (IV) compounds set them apart from platinum (II) compounds. To enable them to achieve their cytotoxic effects, they are often created as prodrugs that are reduced to the active platinum (II) form in cancer cells. The resistance to platinum (II) medications can be overcome by improving the drug’s pharmacokinetic features, selectivity, and biological activity by adding different substitutions at axial locations [65]. In recent years, the design of platinum (IV) prodrugs, particularly photochemical platinum-based prodrugs, has demonstrated a wide range of potential applications in drug-resistant gene recognition. Through photoactivation, photochemical platinum-based prodrugs deliver active platinum (II) drugs that can operate selectively at the tumor location and increase drug-resistant cells’ sensitivity [66]. Further, photochemical platinum-based prodrugs’ targeted nature lowers the drugs’ systemic toxicity and may be beneficial for clinical applications.

However, they have not yet received clinical approval, in part due to the fact that they do not selectively activate malignant tissues outside of the tumor.

##### Application of CRISPR Screening Technology in Drug Resistance Gene Discovery

Since its introduction, CRISPR screening technology has grown in significance as a tool for studying tumor treatment resistance. Researchers can systematically find the genetic causes of chemotherapy resistance through genome-wide CRISPR knockdown screening. For instance, Fetten’s group used 30 genome-wide CRISPR knockout tests in 2024 to thoroughly identify genetic determinants of resistance to several chemotherapy medicines, including oxaliplatin, and suggested possible targeting tactics against resistance. The study further highlights PLK4 as a crucial target for overcoming oxaliplatin resistance, emphasizing that resistance may be conquered by pharmacologically inhibiting PLK4 or by genetic ablation [67]. It also indicates that CRISPR screening is a promising technology with significant growth potential.

In conclusion, there are substantial opportunities for CRISPR screening technology and platinum (IV) prodrug design in the search of drug resistance genes. While CRISPR screening technology offers a foundation for developing new therapeutic targets by methodically identifying drug resistance-related genes, platinum (IV) prodrugs offer a novel approach to combating drug resistance by enhancing medication bioavailability and targeting. With the goal of clarifying resistance processes, as well as developing more potent treatment alternatives, future research might consider combining the two.

#### 5.3.2. Prediction of Drug Resistance

The use of machine learning (ML) and artificial intelligence (AI) technologies for drug resistance prediction has grown greatly in recent years. These technologies are capable of swiftly and correctly predicting drug resistance in pathogens by analyzing vast amounts of genomic, transcriptomic, and clinical data. This provides crucial assistance for therapeutic therapy [68].

##### Application Scope of Drug Resistance Prediction Technology Based on Artificial Intelligence Technology

The following areas are the primary focus of the use of AI technology in drug resistance prediction:

(1) Deep research into genomic data: AI can forecast pathogen resistance to drugs using whole genome sequencing (WGS) and machine learning models. As an example, one study predicted antibiotic resistance in multidrug-resistant Acinetobacter baumannii-utilizing deep neural network (DNN) models in conjunction with WGS and gene expression data, with a prediction accuracy of 98.64% [68].
(2) Analysis of mass spectrometry data: AI techniques can also be used to study mass spectrometry data to predict antimicrobial resistance. For instance, by employing machine learning algorithms to evaluate clinical strains’ mass spectrometry data and combining them with data on drug resistance, helpful classifiers like gradient-enhanced decision trees (LightGBMs) and deep neural network classifiers (MLPs) have been developed, which have greatly boosted the accuracy of drug resistance prediction [69].
(3) Integration of multi-omics data: AI models could more accurately forecast drug resistance by integrating data from genomes, transcriptomics, and metabolomics. In this regard, AI models may predict resistance phenotypes and minimum inhibitory concentrations (MICs) by investigating the relationship between bacterial DNA sequences and antimicrobial resistance phenotypes [69].

##### Specific Applications of Artificial Intelligence in Drug Resistance Prediction

Weis et al. created the large-scale database of clinical strains (DRIAMS) by means of mass spectrometry data [69]. It incorporates resistance information for over 70 antimicrobial drugs, as well as mass spectrometry data for more than 300,000 clinical strains. The three following machine learning algorithms were trained by the researchers using these data: logistic regression, a gradient-enhanced decision tree (LightGBM), and a deep neural network classifier (MLP). The findings revealed that MLP and LightGBM were the most efficient methods for predicting resistance, and they had the potential to greatly improve the effectiveness and dependability of antibiotic stewardship. Not only that, clinical drug resistance monitoring and therapy choices are also being made in AI technology. In particular, AI models can forecast medication resistance and offer tailored suggestions for clinical therapy by analyzing a patient’s electronic health record (EHR) and microbiome data [69].

The beneficial effects of oxaliplatin in the treatment of colorectal cancer have been estimated in recent years using machine learning and artificial intelligence techniques. One study, for instance, created a novel machine learning model called the Colon Oxaliplatin Signature (COLOXIS) model to assess clinical and genomic data from patients and calculate how well they would respond to oxaliplatin treatment. These methods can enhance individualized therapy and more precisely forecast a patient’s reaction to treatment by incorporating multi-omics data [70]; however, the model is still in the primary construction stage and can only be used for oxaliplatin in oxaliplatin-responsive patients, requiring further research and optimization.

These automated systems have performed exceptionally well, with accuracy and precision reaching the same level as a trained examiner’s. Additionally, it has been reported that artificial intelligence technology has been widely used in dentistry to predict the need for orthodontic treatment and orthodontic treatment planning by noticing head measurement markers, determining the need for orthodontic extractions, determining the level of cervical spine maturity, and predicting facial attractiveness adhering to orthognathic surgery. These artificial intelligence (AI) tools not only make tasks easier and yield faster results, but they also help dentists save time and carry out their jobs more effectively. This illustrates the potential and worth of AI technology in a medical context [71].

There is a lot of opportunity for growth in the adoption of AI and machine learning technology for drug resistance prediction. As previously stated, AI might successfully anticipate drug resistance and offer crucial support for the development and clinical use of antimicrobial medications by thoroughly analyzing genomes, transcriptomics, and mass spectrometry data. AI will become more involved in drug resistance research and clinical treatment in the future, as multimodal data fusion and reinforcement learning approaches are introduced.

**Figure 4:**
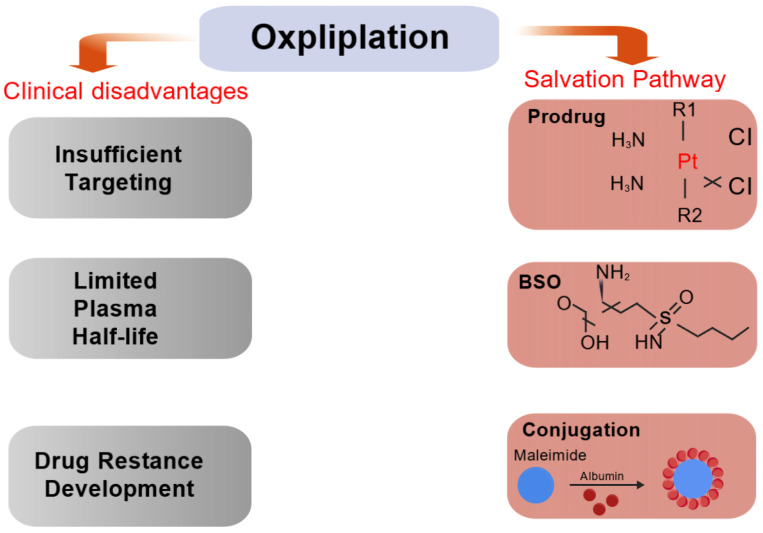
Problems with oxaliplatin in clinical therapy. Created using BioGDP.com [35].

In conclusion, there are still several clinical issues with oxaliplatin therapy, such as its limited plasma half-life, the development of metabolism-based resistance, and insufficient tumor targeting, which results in severe side effects. However, it is expected that these restrictions can be overcome by using oxaliplatin (IV) prodrugs, appropriate resistance modifiers (BSO) as ligands, and conjugation to endogenous natural nanocarriers (albumin) via maleimide (Figure 4).

### 5.4. Utilizing Biomarkers and Therapeutic Targets to Diminish Oxaliplatin Resistance

#### 5.4.1. Targeting Kinases to Conquer Oxaliplatin Resistance

**Figure 5:**
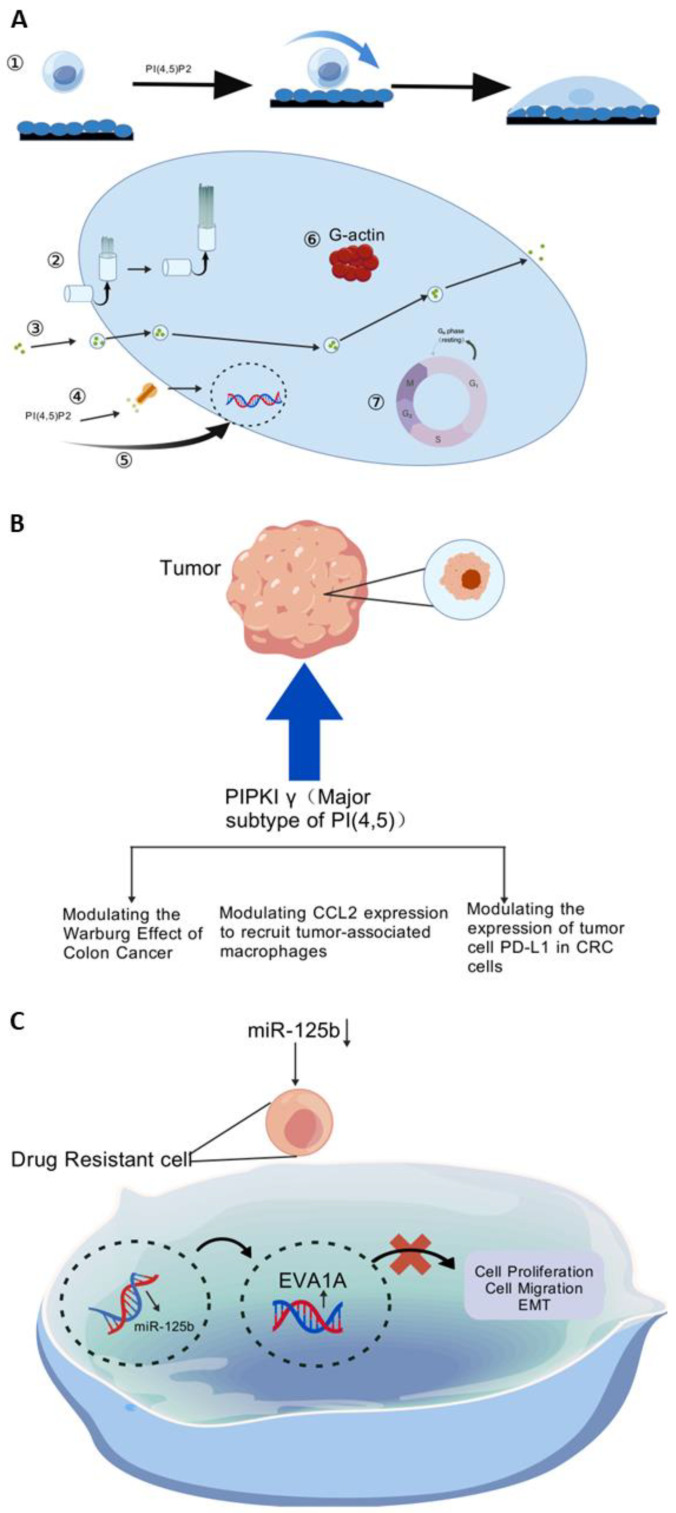
(**A**) A range of cellular activities, in which PI(4,5)P2 is involved as a substrate: ① focused adhesion sssembly; ② cilium formation; ③ vesicular transport; ④ signaling pathway transduction; ⑤ gene expression; ⑥ actin polymerization; ⑦ cell cycle progression. (**B**) The role played by PIPKIγ in tumors. (**C**) Mechanisms of miR-125b effects on drug-resistant cells. Created using BioGDP.com [35].

Patients with colorectal cancer frequently receive therapy with oxaliplatin, a chemotherapy based on a platinum drug [72]. Furthermore, optimizing existing treatment methods for colorectal cancer requires an understanding of the molecular mechanisms underlying oxaliplatin resistance [73]. A variety of differentially expressed genes (DEGs) linked to oxaliplatin resistance were identified through screening and analysis of the RNA sequencing profiles of patients with colorectal cancer receiving oxaliplatin therapy [74]. A comprehensive search was then conducted to determine the differentially expressed genes linked to oxaliplatin resistance, so as to knock down the final group of relevant kinases, the PIPKIs. The enzymes known as PIPKIs are responsible for the synthesis of phosphatidylinositol 4,5 diphosphate (PI(4,5)P2) [75]. PIPKIs can be classified into the following three different isoforms based on their c-terminal sequence: PIPKIα, PIPKIβ, and PIPKIγ. Of those, PIPKIγ is the main isoform that synthesizes PI(4,5)P2. A variety of cellular processes, including focal adhesion assembly, cilium creation, actin polymerization, vesicle transport, cell cycle progression, signaling pathway transmission, and gene expression, can be facilitated by PI(4,5)P2 as a substrate [76,77,78,79] (Figure 5A). PIPKIγ has several functions in malignancies, such as controlling the Warburg Effect in colon cancer [80], recruiting tumor-associated macrophages by modulating the expression of CCL2 [81], and controlling the expression of PD-L1 in tumor cells and colorectal cancer cells [82] (Figure 5B).

Studies on loss-of-function and gain-of-function have shown that PIPKIγ and oxaliplatin resistance are closely related. Furthermore, oxaliplatin resistance was reduced both in vitro and in vivo by pharmacologically blocking the PIPKIγ-exosomal PD-L1 axis. Apart from PIPKIγ, multiple additional genes linked to oxaliplatin resistance were shown to have variable expression. Further studies on these genes may also provide significant information on how to treat colorectal cancer’s oxaliplatin resistance.

#### 5.4.2. Targeting MiRNA to Minimize Oxaliplatin Resistance

The significance of long-stranded non-coding RNAs in controlling drug resistance is becoming more and more clear [83]. Though oxaliplatin-based chemotherapy has recently been shown to be effective at treating advanced HCC, its clinical efficacy is severely limited by the emergence of treatment resistance [84]. A class of tiny non-coding RNAs with 17–25 nucleotides is known as microRNAs (miRNAs). Moreover, miRNAs attach directly to the 3′-untranslated region (UTR) to control gene expression. The crucial significance of aberrantly expressed miRNAs in chemotherapy resistance, especially oxaliplatin, has been confirmed by a growing number of studies in recent years [85,86]. Depending on its target gene, aberrant production of miRNAs can act as an oncogene or tumor suppressor, and it has been linked to the pathogenesis of a number of disorders, including cancer. According to recent reports, a highly conserved miRNA family member, microRNA-125b, has been identified as a hepatocellular carcinoma (HCC) biomarker. It is both resistant to and susceptible to oxaliplatin [84] (Figure 5C).

By preventing cell proliferation, migration, and the epithelial–mesenchymal transition (EMT), miR-125b was downregulated in drug-resistant cells and overexpressed in susceptible cells, which reduced resistance to oxaliplatin, as shown by analysis of HCC cell lines. It demonstrated that oxaliplatin resistance was linked to the downregulation of miR-125b. Additionally, hepatocellular carcinoma patients’ resistance to oxaliplatin treatment was linked to the downregulation of miR-125b, which was mediated via its target, EVA1A [84], thus illustrating how miR-125b regulates its target, EVA1A, to contribute to the possible mechanism of oxaliplatin resistance.

### 5.5. Decreasing Oxaliplatin Resistance by Inducing Anti-Apoptotic Pathway

#### 5.5.1. Inhibition of PLK4

It has been discovered that pharmacologically or genetically blocking PLK4 can overcome oxaliplatin resistance in a variety of animals [67]. Consequently, PLK4 may be a viable therapeutic target for the creation of single-agent approaches to combat drug resistance. One of the main obstacles limiting the effectiveness of treatments for many cancer types has been resistance to chemotherapy. For cancer patients, this obstacle remains a leading cause of treatment failure and disease recurrence [87].

**Figure 6:**
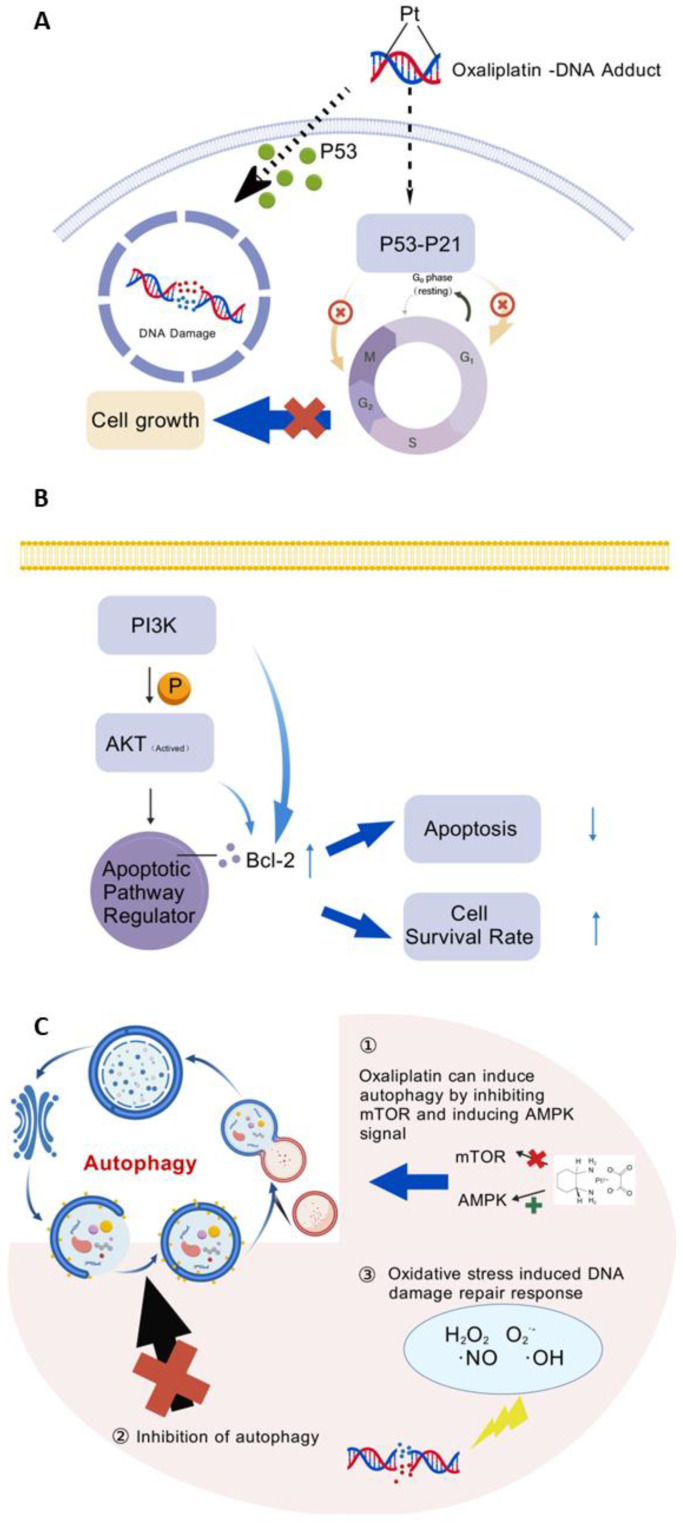
(**A**) Oxaliplatin–DNA adducts block cell growth in oxaliplatin-sensitive cells. (**B**) Hyperactivation of the PI3K/AKT signaling pathway. (**C**) Mechanism of overcoming oxaliplatin resistance by inhibition of autophagy. Created using BioGDP.com [35].

Oxaliplatin–DNA adduct formation results in p53-sensitive DNA damage in susceptible cells. In oxaliplatin-sensitive cells, the activated p53-p21 pathway causes G1 and G2/M cell cycle arrest, which stops cell growth (Figure 6A). Upon specific genetic or epigenetic modifications that inhibit the p53-p21 pathway from functioning (e.g., TP53, CDKN1A, or SLC43A2 knockdown), the cells acquire resistance to oxaliplatin therapy as a result of unchecked cell cycle progression. PLK4 expression is consistently adversely regulated by p53 in response to stress [88,89]. Spindle assembly failure and cytoplasmic division that preferentially occurs in drug-resistant cells might result from genetic ablation or pharmacological inhibition of PLK4 [67]. PLK4 is commonly mutated in human malignancies in a variety of cancer types, including colorectal cancer. In colorectal cancer, tumors with mutant PLK4 typically have a higher tumor mutational burden and are usually associated with advanced tumor progression stages.

#### 5.5.2. Inhibition of Signaling Pathways

Oxaliplatin resistance has been linked to certain signaling pathways, including hyperactivation of the PI3K/AKT signaling pathway [90].

Multiple studies have demonstrated that, after being phosphorylated by PI3K, activated AKT can directly affect apoptotic pathway regulators, such as Bcl-2 family members, and that PI3K/AKT signaling can increase Bcl-2 expression and activity, boosting cell survival and lowering chemotherapy-induced apoptosis [91,92]. Anti-apoptotic and pro-apoptotic proteins, particularly those in the Bcl-2 family, interact to tightly regulate apoptosis, which is a common type of cell death [93]. One profitable sign of whether a cell has undergone apoptosis is the ratio of pro-apoptotic to anti-apoptotic Bcl-2 family proteins [94].

### 5.6. Autophagy

The most often prescribed treatment with chemotherapy drugs for patients with colorectal cancer is oxaliplatin; however, a number of resistance mechanisms, including autophagy, restrict its anticancer effects (Figure 6B). Tumor formation is tightly linked to autophagy, which also raises resistance to negative environmental factors [95]. Radiotherapy/chemotherapy and metabolic stress both trigger autophagy in the tumor microenvironment, which can increase tumor cell resistance to both forms of stimulation [77].

OXA modulates autophagy, which leads to the development of OXA resistance, according to numerous evaluations. For instance, OXA triggers AMPK signaling and inhibits mTOR signaling to cause autophagy [96]. As a platinum medication, OXA prevents transcription and DNA replication by joining with DNA through platinum atoms. DNA is oxidatively damaged by reactive oxygen species (ROS), which are produced by OXA. The latter induces the endogenous apoptotic pathway NA in the mitochondria. It is well known that autophagy contributes greatly to the development of oxaliplatin resistance in cancer cells that are resistant to oxaliplatin [83,97].

#### 5.6.1. Oxidative Stress-Induced DNA Damage Repair Response

Through the autophagy system, oxidative stress-induced DNA damage repair response is a key mechanism for oxaliplatin resistance induction [98]. As the literature reports, oxaliplatin enhances oxidative stress, which in turn causes oxaliplatin-resistant gastric cancer cells to experience less oxidative stress. Oxaliplatin’s primary mechanism of action is attaching to and disrupting DNA, which prevents DNA replication [99] and ultimately has anticancer effects. Among these, autophagy has been connected in the regulation of resistance to oxaliplatin.

Fu et al., for instance, discovered that autophagy activation caused hepatocellular carcinoma to become resistant to oxaliplatin [100]. Additionally, autophagy triggered by the E3 ubiquitin ligase RNF135 was shown to be crucial in causing oxaliplatin sensitization in patients with colorectal cancer [101]. A number of studies have found that the oxaliplatin-associated DNA damage response can trigger autophagy, thus enhancing oxaliplatin resistance [95]. Apoptosis and DNA damage have been linked to oxidative stress.

There is mounting evidence that platinum resistance is mediated in part by oxidative stress. Sen Wang et al. demonstrated that H. pylori infection-induced PRDX2 promoted cisplatin resistance and prevented oxidative stress and double-strand breaks [102]. Oxidative stress is a key factor in gastric cancer, as it mediates the cells’ resistance to oxaliplatin. One major regulator mediating oxaliplatin resistance has been identified as oxidative stress brought on by oxaliplatin treatment. It implies that the DNA damage repair response brought on by oxidative stress is a key mechanism for triggering autophagy and oxaliplatin resistance. The promotion of cellular autophagic flow by oxidative stress has been well-documented [103].

#### 5.6.2. Autophagy Inhibition

Some of the research indicates that autophagy inhibition can reverse and overcome oxaliplatin resistance [95].

Since autophagy is a defense mechanism against ROS, cancer cells have repurposed it as a cytoprotective strategy to combat the genotoxic stress brought on by anticancer medications [96]. To avoid further ROS from being produced, autophagy eliminates oxidized proteins and certain ROS-producing organelles, like mitochondria and peroxisomes. Autophagy inhibitors have now been used to increase the effectiveness of chemotherapy in the treatment of OXA. For example, it has been shown that PFK-15, a small molecule antagonist of PFKFB3, reverses OXA resistance in CRC cells by inhibiting autophagy [104]. Furthermore, in both normal and hypoxic settings, CQ increases the susceptibility of CRC to OXA by acting as an autophagy inhibitor [104].

Autophagy inhibition may be a novel strategy to reverse therapeutic resistance to oxaliplatin, as shown by mounting data [83]. Furthermore, to increase the effectiveness of oxaliplatin, autophagy suppression might be required. Yanli et al., for instance, showed that ATG12-mediated autophagy suppression was necessary for the potentiation of oxaliplatin following WASF knockdown [105]. An essential mechanism for fostering autophagic fusion is the ATG5-ATG12-ATG16L1 complex [106].

We found that oxaliplatin-resistant gastric cancer cells had increased autophagy, indicating that activated autophagy might be a key factor in causing oxaliplatin resistance. We observed reduced apoptosis in oxaliplatin-resistant cells, showing that autophagy may be linked to oxaliplatin-induced apoptosis, which is the primary mechanism by which oxaliplatin suppresses the growth of cancer. More significantly, cellular resistance to certain threats, such as platinum therapy, and eventually to drug resistance, are facilitated by autophagic cell death, which includes autophagy-associated apoptosis [107]. Hence, it is thought to be possible to reverse chemotherapy resistance by focusing on autophagy, as is the case with chloroquine [108]. Moreover, oxidative stress is increased by oxaliplatin treatment. Owing to research conducted from this angle, it has been shown that lowering oxidative stress in tumor tissues may enhance the anticancer effects of platinum medications [109].

### 5.7. Impact of DNA Damage Pathways on Increasing Anticancer Efficiency and Reducing Systemic Drug Resistance

#### 5.7.1. DNA Damage Repair

DNA damage is crucial for lowering systemic resistance and boosting the efficiency of platinum-based anticancer medications. The main way that platinum medications, like cisplatin, carboplatin, and oxaliplatin, prevent tumor cell growth is by generating adducts with DNA that obstruct transcription and DNA replication, which eventually results in cell death. The dependence of oxaliplatin on DNA damage signaling is distinct [110].

Based on reports, one of the main reasons anticancer treatments fail after an initial response is the emergence of acquired resistance. To develop more effective treatment strategies, we must have a deeper comprehension of the mechanisms underlying resistance acquisition. Therefore, it should come as no surprise that platinum-based medications, such as oxaliplatin, are believed to largely work against cancer by destroying DNA [111]. Oxaliplatin is a member of a class of platinum-based cancer treatments that directly interact with DNA to break DNA strands, thus compromising DNA integrity.

Oxaliplatin, a third-generation platinum medication, acts fundamentally from the first two generations of platinum medicines (cisplatin and carboplatin), according to our investigation of Pt–DNA adducts [24]. Furthermore, the cross-resistance profile between oxaliplatin and cisplatin/carboplatin may be lacking, and oxaliplatin may have a better toxicity profile than the two commonly utilized platinum-based anticancer medications, cisplatin and carboplatin [112]. The primary DNA damage repair mechanism brought on by oxaliplatin has been identified as nucleotide excision repair [111]. In this regard, the nucleotide excision repair capacity can be used to determine cellular susceptibility to platinum medicines. Major sensors, signaling, and effector proteins are among the main DNA repair mechanisms [113].

ATR and ATM function as signaling proteins to activate the CHK1 hierarchical linker proteins, which creates favorable conditions for HR repair and recruits effector proteins, like RAD51, for HR repair. In the event of severe DNA damage, such as DNA double-strand breaks, the MRE11-RAD50-NBS1 complex (MRN) can act as a sensor to initiate HR repair [114]. Platinum resistance is far more complicated than we realize, though.

#### 5.7.2. Blocking Homologous Recombination Repair

Massive DNA double-strand breaks (DSBs) and apoptosis can result from oxaliplatin’s ability to generate platinum–DNA adducts, which prevent DNA replication and transcription [113].

One important member of the p21-stimulated serine/threonine protein kinase family [115] is the p21-activated kinase 6 (PAK6) gene, which facilitates the repair of DNA double-strand breaks and mediates tumor chemoresistance through accelerated homologous recombination (HR).

In order to facilitate the activation of ATR, which in turn triggers the downstream repair protein CHK1, PAK6 enters the nucleus. CHK1 then transfers RAD51 from the cytoplasm to the DNA damage site and fixes damaged DNA in GC. By blocking PAK6-mediated HR repair, an ATR inhibitor (AZD6738) can reverse oxaliplatin resistance and even increase susceptibility to the drug [113].

#### 5.7.3. Nucleus Destruction

Pt-induced cytotoxicity is now thought to be primarily caused by Pt adduct buildup on DNA, which activates pro-apoptotic and DNA damage response (DDR) pathways. Studies applying the first-generation medication cisplatin serve as the primary foundation for this paradigm, which is further corroborated by a substantial amount of cellular and biochemical data.

Oxaliplatin’s phenotypic investigations have revealed that it functions better as a transcriptional/translational inhibitor than as a normal DNA-damaging agent. This has prompted researchers to look into alternative targets in an attempt to understand how it works. Oxaliplatin has been demonstrated to be a selective inhibitor of nucleolus transcription, meaning that it causes nucleolus stress and specifically and potently inhibits rRNA synthesis [110].

p53-dependent apoptosis is induced by nucleolus stress. Meanwhile, the early apoptotic signaling following nucleolus stress points to a direct connection between oxaliplatin cytotoxicity and nucleolus stress. Instead of causing significant nucleus–nucleus DNA damage, we discovered that oxaliplatin triggers a unique ATM/ATR signaling pathway that inhibits PoI I transcription, encourages p53 activation, disrupts ribosome synthesis, and eventually results in cell death [110].

**Figure 7:**
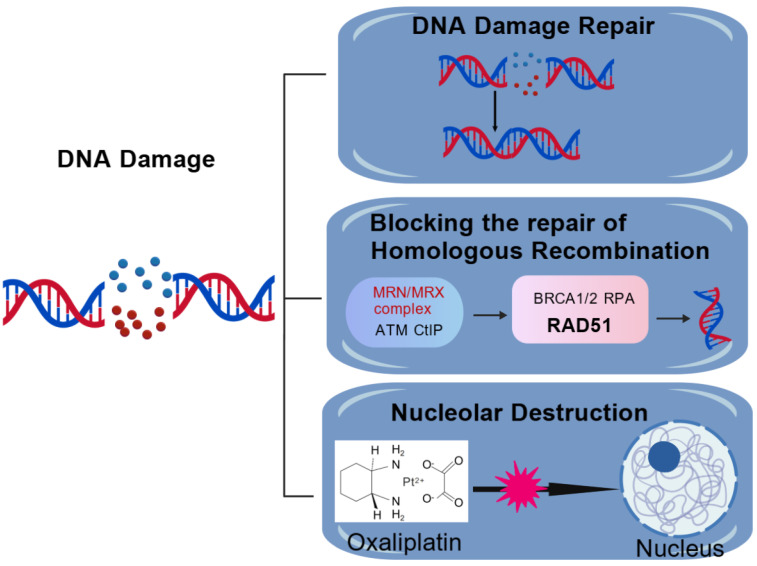
Impact of DNA damage pathways on improving anticancer efficiency and reducing systemic drug resistance. Created using BioGDP.com [35].

Recent research has indicated that oxaliplatin, one of the three FDA-approved platinum medications, is more appropriate as a transcriptional/translational inhibitor, given the fact that platinum compounds were once thought to be DNA-damaging agents [116]. Furthermore, the connection between DNA damage signaling and oxaliplatin’s transcriptional inhibition in the nucleolus should result in the generation of new pathways to lessen oxaliplatin resistance, indicating that oxaliplatin kills cells in a fundamentally different manner than other platinum drugs (Figure 7).

## 6. Combination Therapy

Combination chemotherapy remains the primary therapeutic option for patients with recurrent or unresectable metastatic hepatocellular carcinoma, despite significant advancements in the management of this disease.

Shiitake polysaccharide, a substance that occurs naturally in medicinal plants, has been shown in past research to have strongly synergistic antitumor effects with oxaliplatin on HepG2 cells in vitro and in H22-homozygous mice in vivo [117]. It does this by blocking NF-κB, Stat3, and survivin signaling via the mitochondrial pathway. In addition, shiitake polysaccharides lessen the negative effects of oxaliplatin.

Because of their increased immunogenicity in the tumor microenvironment (TME) and the higher mutational burden of BLCA, immune checkpoint inhibitors (ICIs), exemplified by anti-PD-1 inhibitors, have transformed the treatment of BLCA in recent years. By attracting immune cells, oxaliplatin (OXA), a second-line chemotherapeutic drug used to treat BLCA, may alter the tumor immune microenvironment (TIME). Additionally, the results of oxaliplatin and PD-1 inhibitors for BLCA point to the possible therapeutic benefits of this strategy for BLCA treatment [118].

### 6.1. XL413, a Novel CDC7 Inhibitor

A crucial kinase for the advancement of the cell cycle, cell division cycle 7 (CDC7) regulates the start of DNA replication during the S phase. It forms an active kinase complex with its cofactor, DBF4. The complex activates deconjugase activity and allows DNA unwinding by phosphorylating a number of DNA deconjugase machinery components, most notably the minichromosome maintenance protein MCM2. Therefore, in order to facilitate DNA replication and drive the cell cycle transition from the G1 to S phase, CDC7 performs a crucial regulatory role in activating replication start sites [119]. Meanwhile, the development of a number of malignancies is tightly linked to its activity. Tumor cell cycle arrest, which prevents tumor cell proliferation, can result from its inhibition [120].

In contrast to corresponding normal tissues, it has been demonstrated that CDC7 is overexpressed in a variety of human primary tumors and carcinomas, such as breast, colon, lung, epithelial ovarian, and diffuse large B-cell lymphomas. Additionally, the overexpression of CDC7 is linked to resistance to medications that damage DNA [121].

As CDC7 is frequently overexpressed in a variety of human malignancies, it is a desirable target for cancer treatment.

### 6.2. Oxaliplatin and XL413 Combination Therapy in Cancer

Progress of Oxaliplatin and XL413 Combination Study

Many human primary tumors and cancer cells, such as breast, colon, lung, epithelial ovarian, and diffuse large B-cell lymphoma, exhibit overexpression of CDC7 when compared to matching normal tissues [120]. Oxaliplatin has been utilized extensively in clinical anticancer therapy [122].

In recent years, preclinical or clinical trials have demonstrated the prospective efficacy of combining CDC7 and CDK4/6 inhibitors with chemotherapeutic drugs to treat a range of malignancies [123]. We discovered that, in patients with colorectal cancer, CDC7 expression was linked to tumor recurrence and a poor prognosis.

When Yufeng Chen et al. used siRNA to reduce CDC7 expression, they discovered that this dramatically reduced the formation of colonies in CRC cells treated with oxaliplatin [124]. Crucially, oxaliplatin resistance in CRC cells was overcome by pharmacological inhibition of CDC7 with the CDC7 inhibitor XL413, which additionally avoided tumor ball formation, clone creation, and cell proliferation. The crucial role of CDC7 in colon cancer cells was also demonstrated by the further in vivo verification that the combination of oxaliplatin and XL413 greatly reduced the formation of xenograft tumors. This suggests that targeting CDC7 may be a potentially viable treatment for people with colorectal cancer.

Since there is strong evidence that improving DNA damage repair is closely related to chemoresistance in cancer cells [125], focusing on DNA damage repair to improve tumor response to platinum-based chemotherapy has emerged as a cutting-edge area of chemotherapy research in recent years [126].

CDC7 inhibitor XL413 was reported by Junping Li to improve the chemotherapeutic impact of carboplatin on ovarian cancer cells when administered sequentially after carboplatin [127]. By inhibiting homologous recombination repair activity, the CDC7 inhibitor XL413 may be the mechanism that causes chemotherapy-induced DNA damage to build up more. One of the major genes involved with chemoresistance in high-grade plasma ovarian cancer (HGSOC) is CDC7, according to bioinformatics analysis [128]. It works by controlling the following two vital procedures for tumor survival: DNA synthesis and DNA damage response. Furthermore, by blocking homologous recombination repair activity, the CDC7 inhibitor XL413 proved to increase the accumulation of chemotherapy-induced DNA damage both in vitro and in vivo, thus amplifying the chemotherapeutic effect of carboplatin. Further, in a combinatorial therapy for TP53 mutant hepatocellular carcinoma, Wang et al. discovered that the CDC7 inhibitor XL413 inhibited many gene characteristics linked to DNA repair in TP53 mutant cells [129].

The aforementioned research indicates that oxaliplatin and the CDC7 inhibitor XL413 may be efficient in the treatment of liver, ovarian, and colon cancers; however, these studies are still in the preclinical stage, and additional clinical trials are required to confirm their safety and effectiveness.

## 7. Relationship Between Strategies for Enhancing Platinum Medicines’ Anticancer Activity

### 7.1. Synergies Between the Various Pathways

According to certain reports, platinum (IV) prodrugs, in conjunction with reducing glutathione levels, can simultaneously increase the anticancer activity of oxaliplatin [48]. Second, we found that the combination therapy treatment of oxaliplatin and biomarkers can also diminish resistance in a synergistic manner [129]. Furthermore, by promoting oxaliplatin-induced apoptotic cell death and concurrently blocking the oxaliplatin-induced DNA damage repair pathway, it has been shown that cancer cells can become sensitized to oxaliplatin-induced DNA damage [130]. In a synergistic relationship, oxaliplatin’s anticancer impact was strengthened.

### 7.2. Pathways in Antagonistic Relationship

Under stress, cells adopt autophagy and apoptosis as defense mechanisms against cellular damage. Autophagy may increase drug resistance by eliminating damaged organelles and indirectly triggering the anti-apoptotic pathway, as demonstrated by some studies [131]. Therefore, in order to prevent a resurgence in medication resistance, autophagy suppression may necessitate concurrent inhibition of the anti-apoptotic pathway. In addition to this, we observed that glutathione’s antioxidant properties may lessen the efficacy of DNA damage repair inhibitors [132]; as a result, while making use of the two together, the dose balance must always be carefully maintained.

## 8. Conclusions and Prospects

Chemotherapeutic medications, which are used in traditional tumor chemotherapy, tend to cause severe adverse effects on healthy cells and tissues [125]. Pt-based anticancer medications are crucial for clinical tumor treatment and have good efficacy. Clinical first-line platinum-based anticancer medications, such as cisplatin, represent relatively old medications with well-established molecular processes and therapeutic effects on malignancies. However, platinum-based anticancer medications have severe side effects that severely restrict their use. The most difficult problem with cisplatin and other platinum-based anticancer medications is still systemic toxicity.

For various malignancies, oxaliplatin is a widely used chemotherapeutic drug, and it works by either blocking gene transcription or causing a G2/M phase blockade by forming intra- and interstrand platinum cofactors in DNA. However, systemic treatment of the disease remains a major challenge, and further improvement in the success rate of chemotherapy is prevented by the development of stubborn resistance to oxaliplatin within the course of treatment.

CDC7 inhibitors, such as XL413, have been a breakthrough in the therapeutic treatment of cancer therapy because they can cause tumor cell cycle blockage, which stops the growth of tumor cells [116]. In this study, we used a DNA-damaging agent, oxaliplatin, in conjunction with a new CDC7 inhibitor, XL413, and the combination therapy worked well. As shown in previous research, CDC7 is overexpressed in a large number of primary tumors and cancer cells [117], and this overexpression profile helps the cells withstand chemicals that damage DNA [126].

The following avenues provide future directions and endeavors for us to construct more effective chemotherapeutic strategies and apply them in the clinic:

(1) Glutathione-based
(2) Using biomarkers and therapeutic targets
(3) DNA damage pathway
(4) Combination therapy

Drug targets (such as CDC7 and its inhibitor XL413), drug combination therapies, the phenomenon of resistance in cancer treatment, and ways to mitigate this phenomenon through various pathways are some of the important aspects of drug resistance that are covered in this review, which offers a more thorough investigation of the complex mechanisms of resistance. However, even after much debate, this study fails to offer a thorough explanation of the processes underlying oxaliplatin resistance, especially with regard to its function in the tumor microenvironment. This has led to a more one-sided approach to the strategies proposed to reduce the resistance that arises in oxaliplatin chemotherapy, and future research needs to further explore these areas to fill the gaps in current research.

In light of the previous claim, the limits of the present investigation are reflected in this review’s inability to adequately address the problem of drug resistance, including its function in the tumor microenvironment. To further address therapeutic demands, future research must go deeper into these areas.

Furthermore, there is still conflicting information in some areas, such as the comparatively small number of trials on the CDC7 inhibitor XL413, resulting in it being unclear how it contributes to drug resistance, which may lead to conflicting findings. Future studies should focus more on critically evaluating these contradicting pieces of evidence and further strengthen the analyses’ depth and reliability by thoroughly examining the underlying reasons for the discrepancies (e.g., study design, sample disparities, experimental procedures, etc.). To gain a more thorough understanding of the intricacy of drug resistance mechanisms, we should also incorporate multicenter study designs, standardized experimental procedures, and multilevel analysis of various study data into our research.

This review’s foundation is a methodical search and screening process that guarantees the findings’ reproducibility and transparency. Nevertheless, limitations might still be present, including inadequate search terms or restrictions in database selection. Further investigations could refine the search approach even more to increase the review’s thoroughness and dependability.

## Figures and Tables

**Table 1:** PubMed database search strategies.

PubMed database
#1 Oxaliplatin [Title/Abstract] #2 XL413 [Title/Abstract] #3 CDC7 inhibitor [Title/Abstract] #4 Drug resistance [MeSH Major Topic] #5 Cancer treatment [Title/Abstract] #6 Combination therapy [MeSH Major Topic] #7 #1 OR #2 OR #3 AND #4 #8 #1 OR #2 OR #3 AND #5 #9 #1 OR #2 OR #3 AND #6

**Table 2:** Timeline of significant turning points in the therapeutic use of three generations of first-line Pt drugs.

Generation	Pt Drug	Molecular Structure	Market Time	Listed Country
First	Cisplatin	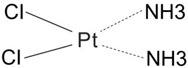	1978	Japan/Italy
Second	Carboplatin	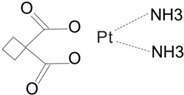	1986	America
Third	Oxaliplatin	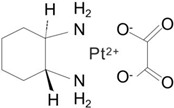	1996	France

## Abbreviations

HCC: hepatocellular carcinoma; HAIC: hepatic artery perfusion; DDK:DBF4-dependent kinase; OXA: oxaliplatin; DACH: diaminocyclohexane; R,R-DACH: cyclohexane-1,2-diamine; GST: glutathione- s -transferase; GSH: glutathione; GCL: glutamate-cysteine ligase; BSO: 1 -butanethionine-s, r—sulfoximine; NAC: thiol n-acetylcysteine; ROS: reactive oxygen species; CRC: colorectal cancer; CQ: chloroquine; MRN: MRE11-RAD50-NBS1 complex; DDR: DNA damage response; ICIs: immune checkpoint inhibitors; CDC7: cytokinesis cycle 7; SCLC: small-cell lung cancer of the lungs; CDK4: cell-cycle regulatory protein kinase 4; CDK6: cell cycle regulatory protein kinase 6; HGSOC: high-grade plasmacytoid ovarian cancer.
